# Loss of loop adenines alters human telomere d[AG_3_(TTAG_3_)_3_] quadruplex folding

**DOI:** 10.1093/nar/gku1245

**Published:** 2014-11-26

**Authors:** Martin Babinský, Radovan Fiala, Iva Kejnovská, Klára Bednářová, Radek Marek, Janos Sagi, Vladimír Sklenář, Michaela Vorlíčková

**Affiliations:** 1 CEITEC—Central European Institute of Technology, Masaryk University, Kamenice 5, CZ-625 00 Brno, Czech Republic; 2 National Centre for Biomolecular Research, Faculty of Science, Masaryk University, Kamenice 5, CZ-625 00 Brno, Czech Republic; 3 Institute of Biophysics, Academy of Sciences of the Czech Republic, v.v.i., Královopolská 135, CZ-612 65 Brno, Czech Republic; 4 Rimstone Laboratory, RLI, 29 Lancaster Way, Cheshire, CT 06410, USA

## Abstract

Abasic (AP) lesions are the most frequent type of damages occurring in cellular DNA. Here we describe the conformational effects of AP sites substituted for 2′-deoxyadenosine in the first (*ap7*), second (*ap13*) or third (*ap19*) loop of the quadruplex formed in K^+^ by the human telomere DNA 5′-d[AG_3_(TTAG_3_)_3_]. CD spectra and electrophoresis reveal that the presence of AP sites does not hinder the formation of intramolecular quadruplexes. NMR spectra show that the structural heterogeneity is substantially reduced in *ap7* and *ap19* as compared to that in the wild-type. These two (*ap7* and *ap19*) sequences are shown to adopt the hybrid-1 and hybrid-2 quadruplex topology, respectively, with AP site located in a propeller-like loop. All three studied sequences transform easily into parallel quadruplex in dehydrating ethanol solution. Thus, the AP site in any loop region facilitates the formation of the propeller loop. Substitution of all adenines by AP sites stabilizes the parallel quadruplex even in the absence of ethanol. Whereas guanines are the major determinants of quadruplex stability, the presence or absence of loop adenines substantially influences quadruplex folding. The naturally occurring adenine-lacking sites in the human telomere DNA can change the quadruplex topology *in vivo* with potentially vital biological consequences.

## INTRODUCTION

Cellular deoxyribonucleic acid (DNA) is constantly subjected to exogenous and endogenous damaging events, which result in the formation of lesions of the building blocks, mainly of the bases (1). Among these lesions, the abasic (AP) sites are the most frequent ones: tens of thousands of purine bases are released per day in each cell, eventuating in one AP site in 10^5^ bases (1,2). The AP site is formed by the spontaneous hydrolysis of the N-glycosidic bond of a nucleotide, by an exposure to irradiation or reactive oxygen species, and also arises as an intermediate in the enzymatic repair processes that remove other carcinogenic base lesions (1). To maintain structural and functional integrity of the genetic material, the majority of various lesions are repaired by the always-alert repair mechanisms. AP sites that are not repaired in time, i.e. before replication or transcription, can be mutagenic, carcinogenic or lethal (1,3). Outcome of the natural and synthetic AP lesions on the stability (4–7), conformation (7–9), repair and replication (6,10,11) of the canonical, double-stranded oligodeoxynucleotides has been widely studied. Non-canonical DNA models—the loop structures formed by a trinucleotide sequence of cytosine, adenine and guanine (CAG) trinucleotide repeat sequence (12,13) and the different types of DNA quadruplexes (14), were also investigated, in most cases using the stable synthetic tetrahydrofuranyl AP sites incorporated in place of nucleosides in selected sequence positions.

Recently, G-quadruplexes, the non-canonical four-stranded DNA structures, have been extensively studied, as G-rich sequences were found in important segments of eukaryotic and prokaryotic genomes (15). They frequently occur in promoter regions of genes, including oncogenes, and participate in processes controlling their expression (16). Quadruplex formation in the telomeric regions inhibits the function of telomerase, which is active in most cancer cells (17). Considering the important role of telomeres in the maintenance of genome integrity and cell survival (18), non-repaired lesions in the overhang structures can have critical consequences *in vivo*.

The sequence integrity of the G tracts is a key factor for G-quadruplex functionality. Four out of the seven published papers on AP site-containing quadruplexes studied the guanine AP sites of the guanine tetrads. These included the tetramolecular parallel quadruplex composed of four 5′-d[T(G)*_n_*T] strands, where AP tetrads were formed (19), the human telomere sequences of 5′-d[G_3_(TTAG_3_)_3_], *htel-21*, by Skolakova *et al.* (20), the d[AG_3_(TTAG_3_)_3_], *htel-22*, by Fujimoto *et al.* (21) and the d[TAG_3_(TTAG_3_)_3_], *htel-23*, by Virgilio *et al.* (22). The sequences contained a single AP site in each G-position. In addition, *htel-22* structure with missing guanines at various positions was also investigated under cell-mimicking conditions using PEG-200 (21). Thymine AP sites were introduced into the 5′-T of d[TGGGT] to study their effect on conformation of sequences with inverted polarity within their molecule, such as 5′-AP-3′-3′-GGGT-5′ and 3′-AP-5′-5′-GGGT-3′, by Esposito *et al.* (23). AP sites in loops have also been investigated. Rachwal *et al.* (24) replaced the thymines by an AP site in the three single-base loops of the parallel quadruplex formed by 17-mer d[TG_3_TG_3_TG_3_TG_3_T]. Beckett *et al.* (25) incorporated 8-oxoguanine and a guanine AP site into the 27-mer nuclease hypersensitive element III_1_ of the human *c-myc* proto-oncogene. This sequence can form two types of coexisting parallel G-quadruplexes. Interestingly, structural transitions between the two quadruplexes were induced and modulated by selection of the location and type of the lesion.

**Figure 1.**
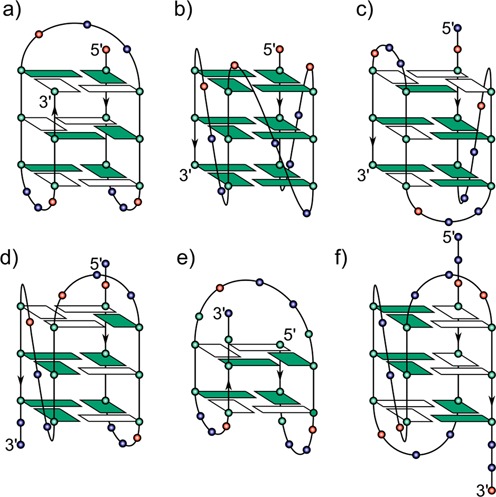
Various quadruplex folding topologies observed experimentally along with the *htel* analogs exhibiting the fold: (**a**) basket-type with edgewise-diagonal-edgewise loop configuration formed in Na^+^ solution (27), (**b**) parallel topology with all loops in propeller-like configuration observed in crystal containing K^+^ ions (28), (**c** and **d**) hybrid-1 (with propeller-like loop followed by two edgewise loops) (29–31,33) and hybrid-2 (two edgewise loops followed by propeller-like loop) (32,33) quadruplex topology observed in K^+^ solution by NMR spectroscopy, (**e**) 2-tetrad basket with edgewise-diagonal-edgewise loop configuration discovered by NMR in K^+^ solution (34,35), (**f**) (2 + 2) quadruplex with edgewise-propeller-edgewise loop configuration recently determined in Na^+^ solution by NMR spectroscopy (36). The positions of deoxyadenosine, deoxythymidine, and deoxyguanosine residues are shown by red, blue, and green circles, respectively. Tetrad deoxyguanosines in *anti*- and *syn*-conformation are represented by green and white rectangles, respectively.

The most frequently studied quadruplex structures are those originating from the 3′-single stranded overhang sequences of the human telomere repeat (TTAGGG)*_n_*. Fragments of this sequence used in structural studies show considerable conformational polymorphism (reviewed in (26)), with a number of quadruplex topologies, which depend primarily on the type of counterions, ionic strength, oligonucleotide sequence, and DNA concentration (see Figure 1) (27–36). For structural studies, a single quadruplex conformation can be induced by altering the 5′- and 3′- terminal oligonucleotide sequences (26,32,33) or by introducing a specific pattern of the *syn-* residues in the quadruplex stem by insertion of 8-bromo-deoxyguanosine (37,38). A single quadruplex conformation can be also achieved by using modified nucleosides which prefer *anti-* conformation of the glycosidic bond, namely 2′-deoxy-2′-fluoro-guanosine and 2′deoxy-2′-fluoroarabinoguanosine (39). Moreover, both the stability of the quadruplex and its topology can be modulated by altering the length of the loop regions connecting individual strands in the G4 stem (40–47). Still, the exact architecture of the telomeric 3′ overhang remains a matter of debate.

**Figure 2.**
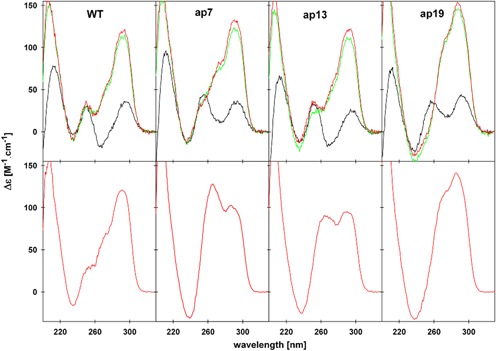
CD spectra of *htel-22 WT* and its AP analogues. Spectra (upper panel) measured at 23 μM DNA strand concentration in 1 mM sodium phosphate and 0.3 mM EDTA, pH 6.8 (black line), and in the presence of 15 mM (green line) and 165 mM (red line) K^+^ ions, respectively, pH 6.9 at 23°C; (bottom panel) measured with the DNA samples at strand concentrations of 0.75–0.89 mM, used for NMR measurements, in 100 mM K^+^, pH 7, at 23°C. The CD spectra measured in 30 mM K^+^ concentration (not shown) were virtually identical to those in the bottom panel.

Though many studies on AP lesions in quadruplexes have been carried out, there is no report on the conformational consequences of their presence on the molecular structure of an intramolecular quadruplex. Using circular dichroism (CD) and nuclear magnetic resonance (NMR) spectroscopies, we have studied the effect of adenine AP site lesions in the TTA loops on the folding of the three-loop quadruplex formed by the human telomere sequence d[AG_3_(TTAG_3_)_3_] in potassium solution. Under our experimental conditions (∼1 mM DNA strand, 30–160 mM K^+^), the natural sequence, *htel-22 WT*, forms a mixture of hybrid-1 (29–31,33) and hybrid-2 (32,33) quadruplexes (Figure 1c and d), which both have two edgewise and one propeller loop and differ in the order of the loops with respect to the oligonucleotide sequence (33). In addition, 3-tetrad (27) (Figure 1a) or 2-tetrad (34,35) (Figure 1e) basket-type quadruplexes having edgewise-diagonal-edgewise loop configuration is also present (48) under the experimental conditions. In this paper, we show that an adenine AP site, in response to its position in the respective loop, substantially changes the polymorphism of the quadruplex arrangement. We have characterized the quadruplex fold of the *htel-22* variants with the AP site in the first and the third loop regions, respectively, and also identified the basis of quadruplex folding dictated by the loss of the adenine base.

## MATERIALS AND METHODS

### Oligonucleotide synthesis

Both the unlabeled and the 1.5% site-specifically labeled oligonucleotides were prepared by an Expedite 8900 oligo synthesizer at the Proteomics Core Facility, Masaryk University, Brno, in 1 micromole scale using Glen Research reagents. Fully (^13^C, ^15^N)-labeled guanine phosphoramidite used for site-specific isotopic enrichment was purchased from Euriso-Top (Saint Aubin Cedex, France). The abasic sites were generated by incorporating the dSpacer CE phosphoramidite, which resulted in a stabilized AP site in the oligonucleotide. Oligonucleotides were purified by Reverse Phase-High Presure Liquid Chromatography (RP-HPLC), and desalted twice by size exclusion chromatography on Sephadex G-25 (NAP25, GE Healthcare). The purity as for the length homogeneity was checked by denaturing electrophoresis (20% gel with 6.2 M urea running at 50°C for 1 h at the power output of 25 W).

### CD spectroscopy and PAGE

A stock solution of about 1000 OD/ml was prepared by diluting the lyophilized oligonucleotides in 1 mM sodium phosphate and 0.3 mM ethylenediaminetetraacetic acid (EDTA) at pH 7.0. Precise DNA strand concentrations were determined on the basis of ultraviolet (UV) absorption at 260 nm of the sample measured in the same low salt solution at 90°C, using molar extinction coefficients of 236 900, 233 100 and 224 300 M^−1^cm^−1^ calculated (49) for d[AG_3_(TTAG_3_)_3_] and its one and three AP sites containing analogs, respectively. UV absorption spectra were measured on a UNICAM 5625 UV/VIS spectrometer (Cambridge, UK).

CD measurements were carried out in a Jobin-Yvon CD6 (Longjumeau, France) and Jasco 815 (Tokyo, Japan) dichrographs in 1-cm to 0.01-cm path-length quartz Hellma cells placed in a thermostated cell holder at 23°C. Scan rate was of 0.5 nm/s. A set of three scans was averaged for each sample. CD signal was expressed as the difference in the molar absorption, Δϵ, of the left- and right-handed circularly polarized light, molarity being related to DNA strands. Before the CD measurements, the DNA samples were heated in the relevant solution for 3 min at 90°C and slowly annealed over the course of 4 h to room temperature. Ethanol dependences were measured by gradually adding 96% ethanol to the DNA samples in 1 mM sodium phosphate and 0.3 mM EDTA, and the resulting DNA concentration was corrected for the volume increase. All CD measurements were performed at room temperature.

UV absorption melting curves were measured in a UV/VIS spectrophotometer (Varian Cary 4000, Mulgrave, Victoria, Australia) from 20 to 98°C and back. The temperature was increased/decreased by 1°C steps and the samples were equilibrated for 2 min before each measurement.

Native polyacrylamide gel electrophoresis (PAGE) was run in a temperature-controlled electrophoretic apparatus (SE-600, Hoefer Scientific, San Francisco, CA). Gel concentration was 16% (29:1 monomer to bis ratio, Applichem, Darmstadt, Germany). About two micrograms of DNA was loaded on the 14 cm × 16 cm × 0.15 cm gel. Samples were electrophoresed at 20°C for 19 h at 30 V. The gel was stained with Stains All (Sigma, St Louis, MO) after the electrophoresis and scanned using a Personal Densitometer SI, model 375-A (Molecular Dynamics, Sunnyvale, CA).

### NMR spectroscopy

Samples for NMR spectroscopy were prepared by dissolving the oligonucleotides in 250 μl of buffer containing 20 mM KH_2_PO_4_/K_2_HPO_4_ in 90%/10% H_2_O/D_2_O with pH adjusted to 6.8. The solutions were annealed by heating to 90°C and cooling at room temperature overnight. The resulting samples of approximately 0.8 mM strand concentrations were transferred into NMR tubes. Samples for measurement in D_2_O were prepared by repeated freeze-drying and dissolving the oligonucleotide in 99% D_2_O. Final sample was prepared by dissolving the pellet in 99.996% D_2_O.

Conventional and ^15^N-filtered 1D spectra in H_2_O, as well as 40 and 80 ms TOCSY and (^1^H, ^13^C)-HSQC spectra in D_2_O, were recorded on a 600 MHz Bruker Avance III spectrometer equipped with a triple-resonance (^1^H, ^13^C, ^15^N) cryoprobe. 2D nuclear Overhauser effect spectroscopy (NOESY), heteronuclear multiple bond correlation with jump-return water suppression (JRHMBC) (50), refocused HNC (51), and long-range ^1^H–^13^C HSQC (51) spectra were recorded on a 950 MHz Bruker Avance spectrometer equipped with a triple-resonance (^1^H, ^13^C, ^15^N) cryoprobe and on 700 MHz Bruker Avance spectrometer equipped with a room-temperature triple-resonance (^1^H, ^13^C, ^15^N) probe. water suppresion by gradient-tailored excitation (WATERGATE) (52) pulse sequence was used for water suppression in all experiments except for the JRHMBC. All spectra were recorded at 25°C. Data were processed using TOPSPIN 3.1 software and 2D NMR spectra were analyzed using program Sparky (53).

## RESULTS AND DISCUSSION

### CD and NMR spectra reveal the influence of abasic lesions on the conformational equilibrium of the K^+^-stabilized d[AG_3_(TTAG_3_)_3_]

To study the influence of an adenine AP site on the structure of the human telomeric sequence d[AG_3_(TTAG_3_)_3_] (*htel-22*), the wild-type (*WT*), three abasic oligonucleotides (*ap7*, *ap13* and *ap19*), and the oligonucleotide containing AP sites in all three loops (*ap7*,*13*,*19*) were prepared (see Table 1).

**Table 1.** Oligonucleotide sequences investigated in this work.

Sequence (5′-to-3′)	Abbreviation
d[AGGGTTAGGGTTAGGGTTAGGG]	*WT*
d[AGGGTT**S**GGGTTAGGGTTAGGG]	*ap7*
d[AGGGTTAGGGTT**S**GGGTTAGGG]	*ap13*
d[AGGGTTAGGGTTAGGGTT**S**GGG]	*ap19*
d[AGGGTT**S**GGGTT**S**GGGTT**S**GGG]	*ap(7,13,19)*

Letter S represents the position of dSpacer mimicking the abasic lesion.

First, the sequences were studied by CD spectroscopy (Figure 2, upper panel). All three 22-mers containing one AP site provided CD spectra corresponding to unstructured forms in 1 mM sodium phosphate and 0.3 mM EDTA. The spectra in 10 mM potassium phosphate buffer (15 mM K^+^) and upon addition of 150 mM KCl (sum of 165 mM K^+^) at pH 6.9 were very similar. The spectrum of the *WT* (Figure 2), dominated by the positive CD signal at 290 nm and a slight shoulder on its short-wavelength site, is characteristic of the K^+^-stabilized human telomere quadruplex (54). On the basis of a non-cooperative course of its formation upon addition of K^+^ ions to the Na^+^-stabilized basket type quadruplex (27), it was suggested (55) that the dominant quadruplex arrangement responsible for the spectrum has the same topology. This is in line with the results of other techniques using relatively low DNA concentrations ((55) and references therein).

Surprisingly, the shoulder on the main 290 nm CD band became more pronounced for the sequences containing an AP site, namely *ap7* and, especially, *ap19*. The increase in intensity of the shoulder indicates structural changes opposite to those caused by the naturally occurring G lesions in the quadruplex core. The guanine AP sites (20), the 8-oxoguanine (56), or the A for G substitutions (57,58) weakened the K^+^-stabilized quadruplex arrangement and additionally shifted the conformational equilibrium toward antiparallel quadruplexes as indicated by the local CD minimum at 260 nm instead of the positive shoulder in their CD spectra. In contrast, the increase of the shoulder at 260 nm observed with the adenine AP sites containing *htel-22* analogues indicates a shift of the quadruplex conformational equilibrium toward higher population of parallel strands (parallel or 3 + 1 quadruplex structures). The thermal stability of *ap13* was decreased compared to *WT* whereas the stability of *ap*7 was not substantially affected and *ap19* was even slightly more stable than *WT* (*T*_m_ values in 165 mM K^+^ were 71.2, 70.2, 66.4 and 73.3°C for the *WT*, *ap7*, *ap13* and *ap19*, respectively, see Supplementary Figure S1a). Native polyacrylamide electrophoresis proved that none of the adenine AP sites disturbed the ability of the *htel* sequences to fold into intramolecular quadruplexes (Supplementary Figure S2a).

**Figure 3.**
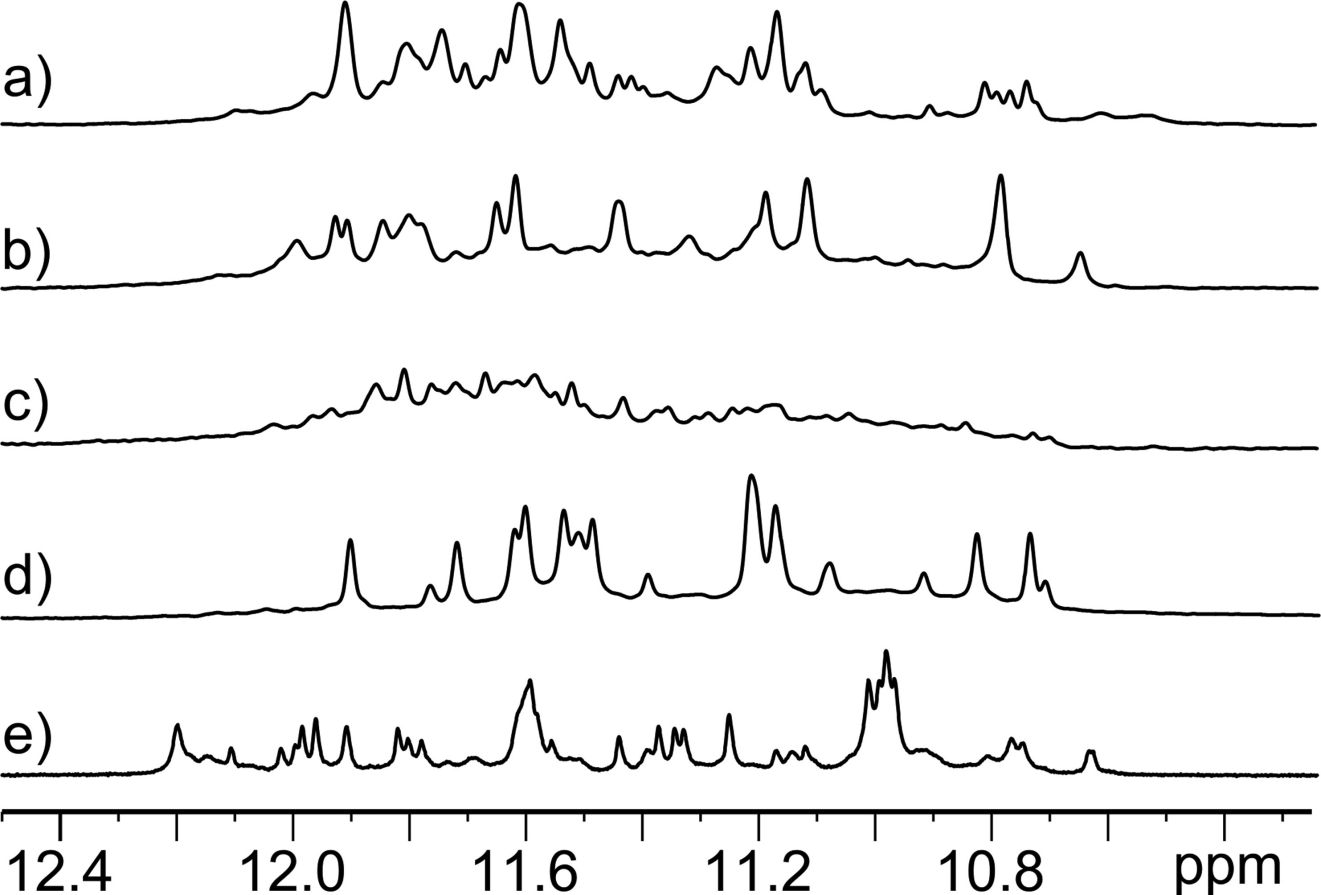
Imino proton regions of 1D NMR spectra of (**a**) *WT*, (**b**) *ap7*, (**c**) *ap13*, (**d**) *ap19* and (**e**) *ap(7,13,19)*. Spectra were measured in 30 mM K^+^, pH 6.8 at 25°C, and ∼0.8 mM DNA strand concentrations.

Further details of the effect of the missing base on the quadruplex structure were elucidated by NMR spectroscopy. The differences in the structural behavior of the *WT* and its AP variants are clearly indicated by the number and line widths of the signals in the imino region of the ^1^H NMR spectra (Figure 3).

The broad and poorly resolved lines in the spectrum of *ap13* indicate that depurination at this position results in an even more heterogeneous mixture of quadruplex forms than in the case of *WT* (Figure 3). Interestingly, the spectra of *ap7*, and especially *ap19*, are much simpler and the number of major signals observed indicates a dominant presence of a single quadruplex arrangement in the solution (Figure 3). Figure 3 thus clearly shows that the position of the abasic lesion influences significantly the equilibrium between the quadruplex conformations. The spectra did not change upon an increase in concentration of K^+^ ions (for *WT* and *ap19*, see Supplementary Figure S3).

Using CD spectroscopy, we have recently shown (55) that folding of the *WT htel-21* and *htel-22* quadruplexes depends on the strand concentrations of the DNA above 2 mM (59). Since the NMR measurements were carried out at DNA concentrations about 40 times higher than the CD spectra shown in the upper panel of Figure 2, we have also measured the CD spectra of the samples used in the NMR study. The results are shown in the bottom panel. The CD spectrum of the 890 μM *WT* sample hardly differs from that measured at low (23 μM) DNA concentration (compare the upper and bottom spectra in Figure 2). However, at the high DNA concentration the spectrum of *ap7* showed substantial increase in the 260 nm signal indicating increasing formation of (3 + 1) and/or parallel quadruplexes, as compared to the low concentration sample. The two positive bands at 260 and 295 nm of comparable heights of the *ap13* may follow from the increased populations of various quadruplex structures, as it can be inferred from the NMR spectra. The CD spectrum of *ap19* did not change qualitatively upon the increase of DNA concentration, indicating that the AP site at the 3′-end loop strongly stabilized a clearly defined topology in K^+^ solution, even at low DNA concentrations. The *T*_m_ values of the DNA samples used for NMR measurements (∼0.8 mM DNA strand and 100 mM K^+^ concentrations) were 69.3, 71.0, 65.9 and 70.7°C for the *WT*, *ap7*, *ap13* and *ap19*, respectively (Supplementary Figure S1b). Again, only the AP site in the middle loop lowered the melting temperature of the *htel-22* quadruplex, whereas a slight increase in *T*_m_ was observed for sequences with the AP sites in the first and third loops as compared with the *WT* ones. The presented results show that the AP site in the outer loops of *htel-22*, and especially in *ap19*, stabilizes a single quadruplex conformation. Therefore, both *ap19* and *ap7* were chosen for a more detailed NMR study in order to elucidate their quadruplex arrangement.

### Assignment of NMR resonances and determination of *syn*-guanines in *ap19* and *ap7*

The determination of quadruplex topologies adopted by *ap7* and *ap19* started with the assignment of H_imino_, H8 and H1′ resonances. Since the assignment strategy was the same for both abasic sequences, we demonstrate our approach using mainly spectra measured for *ap19*. Figures documenting the resonance assignment of *ap7* can be found in the Supplementary Information.

**Figure 4.**
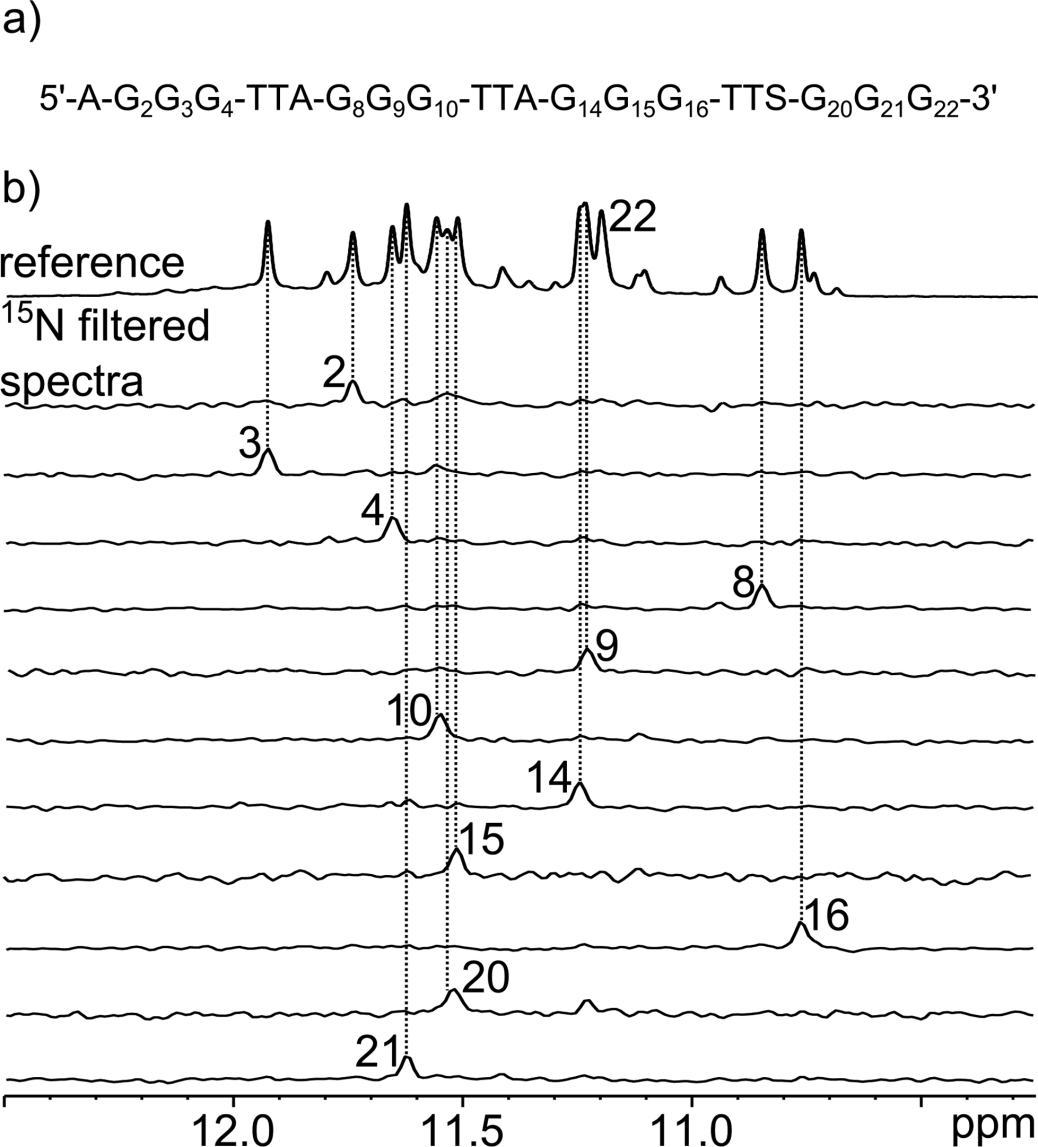
(**a**) Sequence of *ap19* with numbered guanine residues. Letter S denotes the position of the dSpacer mimicking the AP site, (**b**) reference proton 1D spectrum of *ap19* and ^15^N-filtered spectra of site-specifically enriched samples used for unambiguous assignment of guanine imino proton resonances. The spectra were recorded at 25°C, pH 6.8 and 30 mM K^+^ concentration.

**Figure 5.**
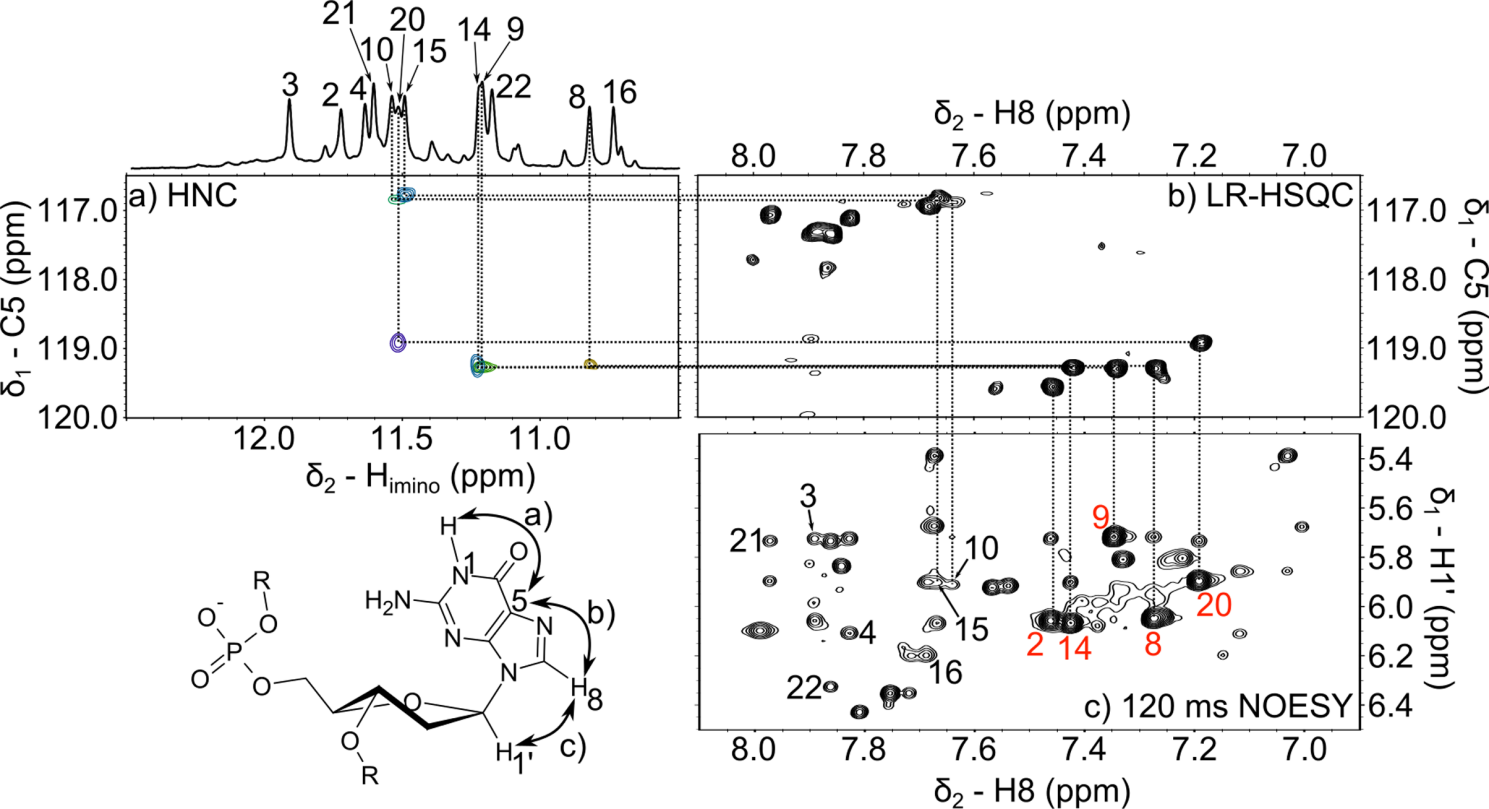
Assignment of H8 and H1′ protons in selected guanine residues of *ap19* (see Figure 4a for the oligonucleotide sequence) *via* heteronuclear correlations to imino protons and NOESY spectra. The numbers in (**c**) denote the assignment of intraresidual H1′–H8 crosspeaks to individual guanosines. Guanosine residues in *syn-*conformation are labeled in red. The H_imino_–C5 crosspeak corresponding to guanine 2 was assigned in JRHMBC spectrum (not shown). The spectra were recorded at 25°C, pH value of 6.8 and 30 mM K^+^ concentration.

Imino ^1^H resonances were successfully assigned using a site-specific (^13^C, ^15^N)-labeled isotopic enrichment (60) of individual guanine residues (see Figure 4 and Supplementary Figure S4 in Supplementary information). The connection between the imino (N1-H) and H8 resonances was confirmed by exploiting the relatively large values of ^3^*J* coupling constants of both protons to carbon C5. Since JRHMBC experiment at natural abundance (50), which is usually used for obtaining such through-bond correlations, was in our case plagued with severe signal overlap, we employed a two-stage strategy using a long-range HSQC and a 2D version of HNC experiment (51) (see Figure 5).

First, the correlation between guanine imino protons and C5 carbons was obtained by measuring the 2D HNC experiment for each site-specifically labeled sample (step a in the bottom left part of Figure 5). The H8 protons were then assigned using a long-range HSQC experiment optimized for observation of H8–C5 correlations (step b). This procedure, while time demanding, enabled us to unambiguously assign each H8 proton of individual guanine residue. In the last step, the analysis of the NOESY spectra revealed intraresidual H1′–H8 connectivities (step c) and identified *syn-*guanines based on the strong H1′–H8 crosspeaks (distance between these protons in *syn*-conformation is approximately 2.5 Å in contrast to that around 3.8 Å for the *anti*-conformation). The complete strategy for the assignment of guanine resonances is shown in Figure 5 for *ap19* and in Supplementary Figure S5 for *ap7*. In the cases of significant overlaps of C5 resonances in LR-HSQC spectra, the assignment of guanine residues was further confirmed by observing H8 signals filtered by ^2^*J*_H8N7_ long-range couplings in the spectra of oligonucleotides with isotopically labeled guanosines (see Supplementary Figure S6 for *ap7* and Supplementary Figure S7 for *ap19*).

Once all the intramolecular H1′–H8 crosspeaks were identified in the NOESY spectrum, we proceeded to assign the loop residues using the standard sequential assignment procedure with additional information from TOCSY (identification of thymidines based on the observable intraresidual CH_3_-H6 crosspeaks) and base-optimized HSQC (identification of H8 resonances via C8-H8 crosspeaks) spectra. The complete sequential assignment in the H1′–H6/8 region of NOESY spectrum carried out for *ap19* is shown in Supplementary Figure S8. In the case of *ap7* we were able to assign only residues T11–A13 belonging to the middle edgewise loop because of the signal overlap (see Supplementary Figure S9). Nevertheless, the assignment of all guanine residues is sufficient for the elucidation of the quadruplex fold. It is apparent that the sequential walk is broken at expected places, i.e. *anti*-*syn* steps resulting in the distances between neighboring H1′ and H8 protons greater than 5 Å (61). Some additional crosspeaks visible in the spectra arise from minor conformations present in the solution. In the case of *ap*7, the relatively high intensity of the signals belonging to the minor form warranted further investigation (see below).

### NMR results obtained for *ap7* and *ap19* reveal their folding into hybrid-1 and hybrid-2 quadruplex in potassium solution

The folding topology of major quadruplex structures formed by *ap7* and *ap19* was determined on the basis of the intensity of intraresidual H1′–H8 crosspeaks and the H_imino_–H_imino_ and H_imino_–H8 NOE connectivities in the NOESY spectra.

**Figure 6.**
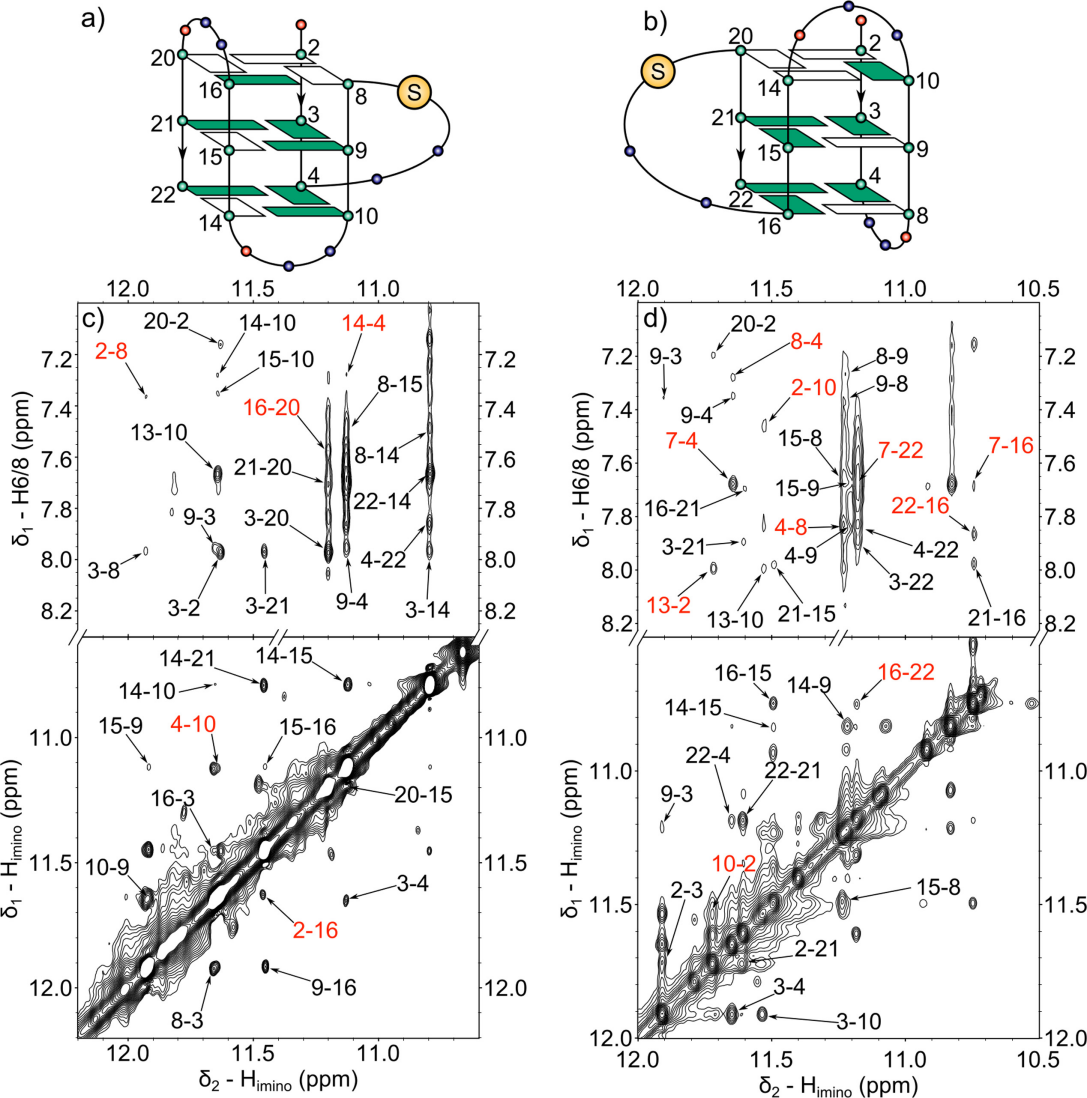
Schematic representations of (**a**) *ap7* (hybrid-1) and (**b**) *ap19* (hybrid-2) quadruplex topologies. Guanines in *syn-* and *anti-*conformation are denoted as white and green rectangles, respectively. Adenines are shown as red and thymines in blue. Large yellow circles denote the positions of AP sites (**c** and **d**) portions of 150 ms NOESY spectra of *ap7* and *ap19*, respectively, showing H_imino_–H_imino_ (lower part) and H_imino_–H8 (upper part) connectivities between bases. The labels denote the positions of the two interacting bases in the sequence. The crosspeaks specific to the particular form of (3 + 1) quadruplex are labeled in red.

The NOESY spectra of both *ap7* (Supplementary Figure S5c) and *ap19* (Figure 5c) displayed five intense H1′–H8 crosspeaks belonging to *syn-*guanines indicating the formation of (3 + 1) quadruplex structures (33). Four of the determined *syn-*guanines (2,8,14,20) were common for both abasic sequences and also for both forms of the (3 + 1) quadruplex which can be distinguished by the position of the remaining *syn-*guanine. In the case of *ap7* the last *syn-*guanine is located at position 15, whereas in *ap19* the remaining *syn-*guanine is located at position 9. These findings indicate that *ap7* folds into the hybrid-1 quadruplex topology comprising of a propeller loop followed by two edgewise loops (Figure 1c), while *ap19* forms the hybrid-2 quadruplex where the first two loops are edgewise and the last one is of propeller type (Figure 1d) (33). To further confirm these initial findings we looked for specific H_imino_–H_imino_ and H_imino_–H8 connectivies in the NOESY spectra of the two abasic sequences (see Figure 6).

Although the majority of the crosspeaks between the guanine imino protons (Figure 6c and d) in both spectra is the same for both (3 + 1) quadruplex topologies, we were able to identify a number of NOE contacts, which can discriminate between the hybrid-1 and hybrid-2 folds. Among these are the NOEs between imino protons of guanines 2 and 16, and between guanines 4 and 10 observed in the spectra of *ap7* (lower part of Figure 6c). Additional H_imino_–H8 crosspeaks were observed between guanines 2 and 8, 4 and 14, and, finally, between guanines 16 and 20 (upper part of Figure 6c). Thus, guanines 2 and 16 are present in the same (top) tetrad of hybrid-1 quadruplex (Figure 6a), whereas they are located on the opposite ends of the quadruplex stem in the hybrid-2 topology (Figure 6b). Similarly, guanines 16 and 20 are located in the top tetrad of hybrid-1 topology, while the distance between them in hybrid-2 is too large for any NOE transfer to take place. Additionally, guanines 4 and 10 are located in the bottom tetrad of hybrid-1 quadruplex. On the other hand, these residues are located on the opposite ends of G4 stem in hybrid-2 topology. We may thus conclude that these NOE crosspeaks are consistent only with *ap7* adopting hybrid-1 quadruplex topology in potassium solution.

The spectra of *ap7* contained relatively intense signals (Supplementary Figure S6c) indicating the presence of another conformational state. Despite their large linewidths and significant overlap with the signals of the major form, we were able to assign C5 resonances of selected guanine residues (see Supplementary Figure S10). We have identified the *syn*-residues based on the tendency of their C5 carbons to resonate 1–2 ppm downfield from the *anti*-guanines (see the JRHMBC spectra presented in (33,34,36)). The distribution of *syn*-guanines together with the linewidths of the minor signals suggest that these resonances belong to higher-order aggregates formed by the observed hybrid-1 topology.

In the case of *ap19* we have also observed NOE patterns characteristic for one of the (3 + 1) quadruplex topologies, such as H_imino_–H_imino_ crosspeaks between guanines 2 and 10, and between guanines 16 and 22 (see the lower part of Figure 6d). For these residues, the pseudosequential intratetrad crosspeaks between imino- and aromatic H8 protons were observed as well (upper part of Figure 6d). In the hybrid-2 topology these residues are located in the same tetrad (Figure 6b) with readily observable NOE crosspeaks between base protons whereas in the hybrid-1 quadruplex topology guanines 2 and 10 are located in the top and bottom tetrad, respectively (Figure 6a). The same applies to guanines 16 and 22. Additional evidence for the *ap19* hybrid-2 quadruplex topology is the crosspeak between H8 proton of adenine 7 (which is located in an edgewise loop stacked on the bottom tetrad formed by guanines 4, 8, 16 and 22), and imino protons of guanines 4, 16 and 22.

These findings, together with the number and location of *syn*-guanines determined above, indicate that both abasic sequences fold into distinct (3 + 1) quadruplex topologies in the K^+^ solution; ap7 exists predominantly as hybrid-1, whereas ap19 folds into hybrid-2 quadruplex topology.

### Understanding the conformational preferences of *ap7* and *ap19* quadruplex folding

**Figure 7.**
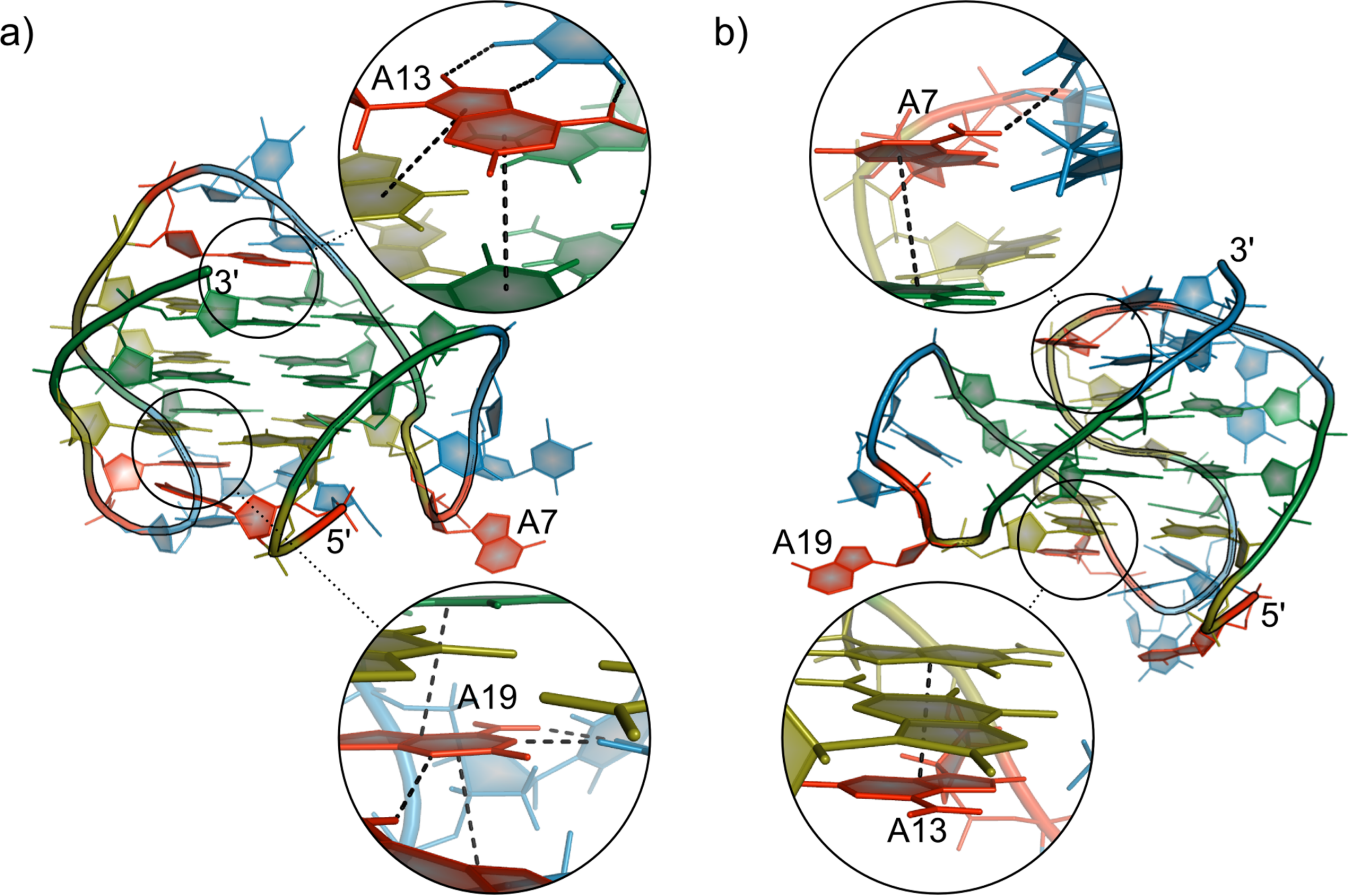
Structures of (**a**) hybrid-1 (PDB ID: *2jsm*), and (**b**) hybrid-2 (PDB ID: *2jsl*) quadruplex topology with detailed view of the adenine bases located in the edgewise loops of both structures. The adenosine residues are numbered according to their position in *WT* sequence. Deoxyadenosine and deoxythymidine residues are colored red and blue, respectively. Deoxyguanosines in *anti*-conformation are colored green, deoxyguanosines in *syn*-conformation around the glycosidic bond are colored dark yellow. The dashed black lines represent the stabilizing hydrogen bonds or the stacking interactions with neighboring residues.

To understand the propensity of *ap7* and *ap19* toward a single quadruplex conformation, we examined the role of adenine bases in the available published structures of various quadruplex folds. (In order to simplify the following discussion we use labels A7, A13 and A19 (Figure 7) to denote adenines in the first, second and third TTA loop in determined quadruplex structures though the primary structure and the length of some of them differed from *WT-22*mer.)

An examination of the hybrid-1 quadruplex structure (see Figure 7a) reveals the origin of the observed effect of the abasic site. In this structure, the first TTA tract forms a propeller-like loop with A7 exposed to the solvent and not participating in any stabilizing interactions with other bases. In contrast, A13 contributes to the stability of middle edgewise loop by forming hydrogen bonds with the preceding thymine and stacking atop the G4 stem. Similarly, A19 stacks on the bottom of G4 stem and one of the loop thymines while forming an extensive hydrogen bonding network with the other loop thymine and 5′ terminal adenine. Depurination of A13 or A19 would thus lead to destabilization of the respective edgewise loops followed by decrease of overall fold stability. On the other hand, depurination of A7 has no dramatic effect on the stability of hybrid-1 quadruplex, since the base is located in the propeller loop.

In the structure of hybrid-2 quadruplex (Figure 7b), the order of TTA loop regions is reversed; the propeller loop is now formed by the TTA region near the 3′ end of the sequence. A7 and A13 stabilize the edgewise loops by forming hydrogen bonds and stacking interactions with other bases, whereas A19 is located in the last propeller loop and does not form any stabilizing interaction with neighbors. Thus, in the case of hybrid-2 topology, depurination of A7 and A13 would destabilize the structure, whereas the loss of A19 has little effect on the stability of the fold, which is indeed the case (Supplementary Figure S1).

It is interesting to note that A13 forms an extensive interaction networks in all K^+^-stabilized quadruplex folds determined so far; depurination of this base would destabilize all of these topologies. The only exception is the 2-tetrad basket topology (Supplementary Figure S11). Our results, however, indicate that *ap13* did not preferentially adopt any of these structures; contrariwise, polymorphism of its quadruplex structures increased (Figure 3).

All the experimental data presented in this work show that, depending on its position (Figure 3), the AP lesion in the loop region of the human telomeric quadruplexes modulates the equilibrium between various quadruplex architectures that coexist in the *WT* solution. In the case of both *ap7* and *ap19* such a lesion results in the preferential formation of a hybrid quadruplex topology in which the AP site is located in the propeller loop connecting parallel oriented neighboring strands. Consequently, in such quadruplex fold (hybrid-1 in the case of *ap7* and hybrid-2 in the case *of ap19*) the negative enthalpic effect of adenine depurination is minimized.

**Figure 8.**
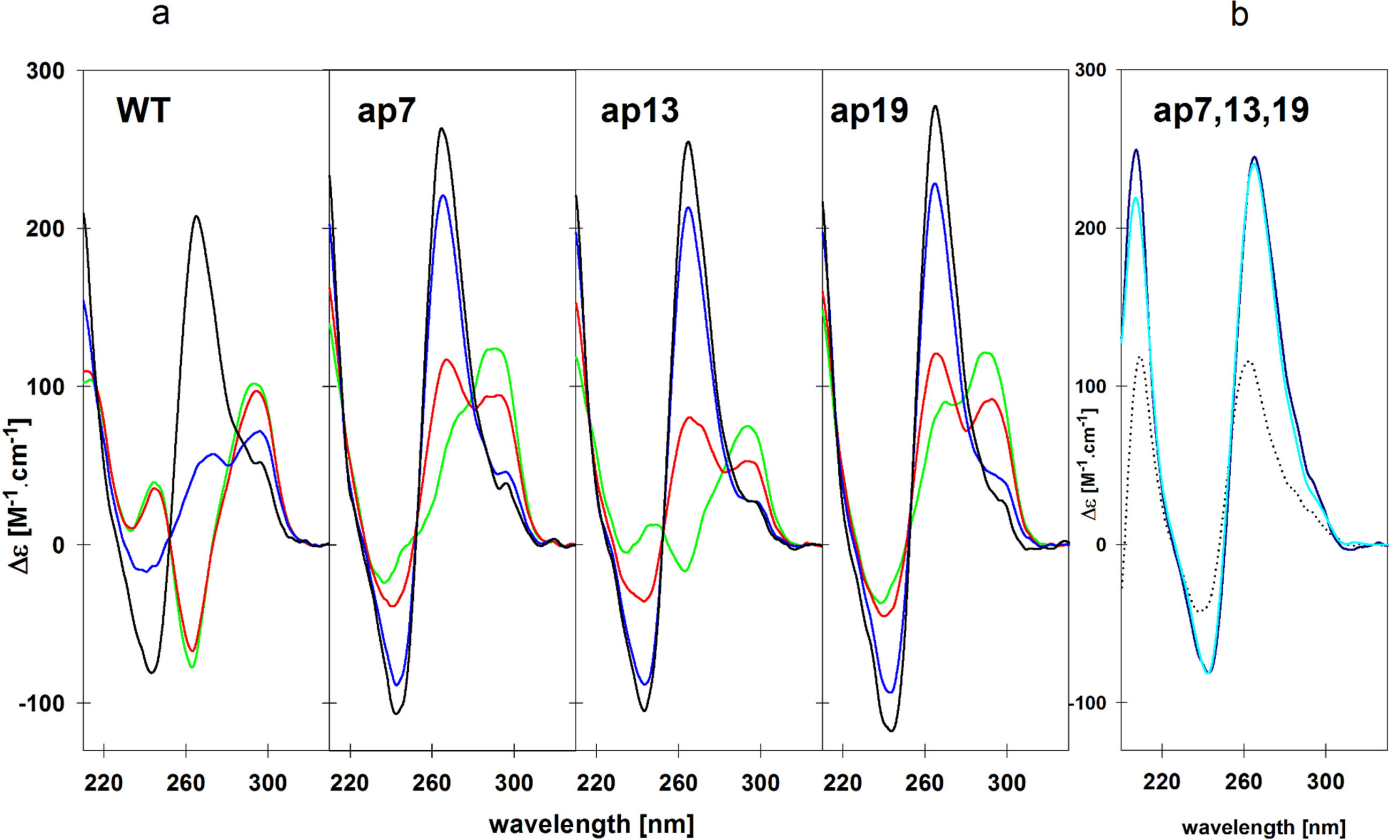
(**a**) CD spectra of *WT* and of its AP analogs in the course of their ethanol-induced transition into parallel quadruplexes. Ethanol concentrations were 30% (green), 50% (red), 57% (blue) plus added 2 mM K^+^ (black). Spectra were measured at 23°C. (**b**) CD spectra of *ap(7,13,19)* measured at room temperature in 1 mM sodium phosphate (black dots), in 20 mM potassium phosphate, pH 6.9 at 0.02 mM (dark blue) and 1.3 mM (light blue) DNA concentrations.

Based on this conclusion it can be assumed that the AP quadruplex containing one propeller loop can be more easily transformed into an all-parallel quadruplex, in which all loops are of propeller-type. The assumption has been confirmed by CD spectra (Figure 8a). All three *htel-22* variants containing an AP site start forming parallel quadruplex under conditions in which the wild type remains in the prevailing antiparallel form and, moreover, they do not need for this conversion K^+^ ions, which are essential for the transition of the *WT* sequence. Thus, the AP site in any loop favors the formation of parallel-stranded motif connected with a propeller loop, which in turn facilitates the refolding of the remaining two loops and the transition toward the all-parallel topology. The *WT* adopts the same parallel quadruplex structure in 57% ethanol and 2 mM K^+^ as the AP *htel-22* sequences, as indicated by the same CD spectrum, but only with long-lasting kinetics or after annealing.

The obtained results led us to examine the type of the quadruplex folding of the *htel-22* sequence containing AP sites in the places of adenines in all three loops. Interestingly, this sequence forms parallel quadruplex even in the aqueous potassium solution regardless of DNA concentration (Figure 8b). The absence of purine base in the loops does not alter the length of the loop backbone and the formed parallel quadruplex remains predominantly intramolecular (Supplementary Figure S2b). The structure does not, however, adopt a single quadruplex fold as it follows from both 1D NMR spectra (Figure 3e) and a fuzzy electrophoretic band (Supplementary Figure S2b).

The loss of tetrad guanines destabilizes the *htel* quadruplex, depending on the position of the lesion, by 10 to 26°C in 0.1 M KCl, and stabilizes the population of antiparallel quadruplexes (20). The present paper demonstrates that the loss of loop adenines does not substantially affect quadruplex stability but effectively alters the type of quadruplex folding by increasing the population of parallel oriented strands in its arrangement.

## CONCLUSIONS

To study the effect of AP sites on quadruplex folding we incorporated APs, one-by-one, in place of each of the three loop adenines of 5′-d[AG_3_(TTAG_3_)_3_] fragment of the human telomere sequence (*htel-22*).

CD spectroscopy and electrophoretic results indicate that the AP site does not hamper the ability of the three modified sequences to fold into intramolecular quadruplexes in potassium solution. However, the equilibrium of quadruplex folds existing in the wild-type sequence is shifted toward higher population of parallel strands in the quadruplex structures at DNA strand concentrations close to 1 mM.

NMR spectra have revealed that the polymorphism of quadruplex arrangements depends on the position of the lesion. While the quadruplex polymorphism increases when the AP site is located in the middle loop, the AP site in the first (*ap7*) and in the third loop (*ap19*) favor a single quadruplex fold. Using NMR spectroscopy, and the site-selective isotopic labeling, we have determined that both *ap7* and *ap19* fold into a (3 + 1) quadruplex, although the exact topology is different for each of these sequences. The sequence with an AP site in the position 7 folds into the hybrid-1 quadruplex, whereas the occurrence of AP site in the position 19 leads to the formation of hybrid-2 topology. Interestingly, the AP site is in both cases located in the propeller-like loop minimizing the potential negative enthalpic effect of the AP site on the stability of the resulting quadruplex structure.

Each of the three adenine AP site-containing *htel-22* variants transforms easier than the *WT* into a parallel quadruplex in dehydrating ethanol solution. Thus, the AP site in any of the loops stabilizes the formation of the propeller loop connecting two parallel strands, which then facilitates the same folding in the remaining loops and formation of the all-parallel quadruplex topology. Introduction of adenine AP sites into all three loops stabilizes parallel quadruplex even in the absence of ethanol in the aqueous potassium solution. The incorporation of adenine abasic lesions into the loop regions of the human telomeric sequence can be used as a powerful tool for manipulating the G-quadruplex structure.

While the loss of tetrad guanines destabilizes the *htel* quadruplex, the loss of loop adenines can change the type of quadruplex folding. Accordingly, the naturally forming adenine AP lesions in the *htel* DNA can change the folding topology of its quadruplexes and shift the conformational equilibrium of its quadruplex structures toward hybrid and all-parallel folding topologies. In view of important functions of the human telomeres in ageing and cancerogenesis, the change in their quadruplex type may have serious biological consequences.

## SUPPLEMENTARY DATA

Supplementary Data are available at NAR Online.

SUPPLEMENTARY DATA
