# Supplementary material for: Loss of loop adenines alters human telomere d[AG3(TTAG3)3] quadruplex folding
Source: Nucleic Acids Res. 2014 Nov 26;42(22):14031–41. doi: 10.1093/nar/gku1245 (PMC4267657; doi:10.1093/nar/gku1245)
Supplement: SUPPLEMENTARY DATA [file supp_42_22_14031__index.html]

Loss of loop adenines alters human telomere d[AG3(TTAG3)3] quadruplex folding — Loss of loop adenines alters human telomere d[AG3(TTAG3)3] quadruplex folding — SUPPLEMENTARY DATA 

# Loss of loop adenines alters human telomere d[AG3(TTAG3)3] quadruplex folding

## SUPPLEMENTARY DATA

**Files in this Data Supplement:**

- SUPPLEMENTARY DATA
