# Supplementary material for: Loss of loop adenines alters human telomere d[AG3(TTAG3)3] quadruplex folding
Source: Nucleic Acids Res. 2014 Nov 26;42(22):14031–41. doi: 10.1093/nar/gku1245 (PMC4267657; doi:10.1093/nar/gku1245)
Supplement: SUPPLEMENTARY DATA [file supp_gku1245_29-10htel22ap_NAR_mb.doc]

**Supplementary Figures for:**

Loss of loop adenines alters human telomere d[AG_3_(TTAG_3_)_3_] quadruplex folding

Martin Babinský, Radovan Fiala, Iva Kejnovská, Klára Bednářová, Radek Marek, Janos Sagi, Vladimír Sklenář^*^, and Michaela Vorlíčková^*^


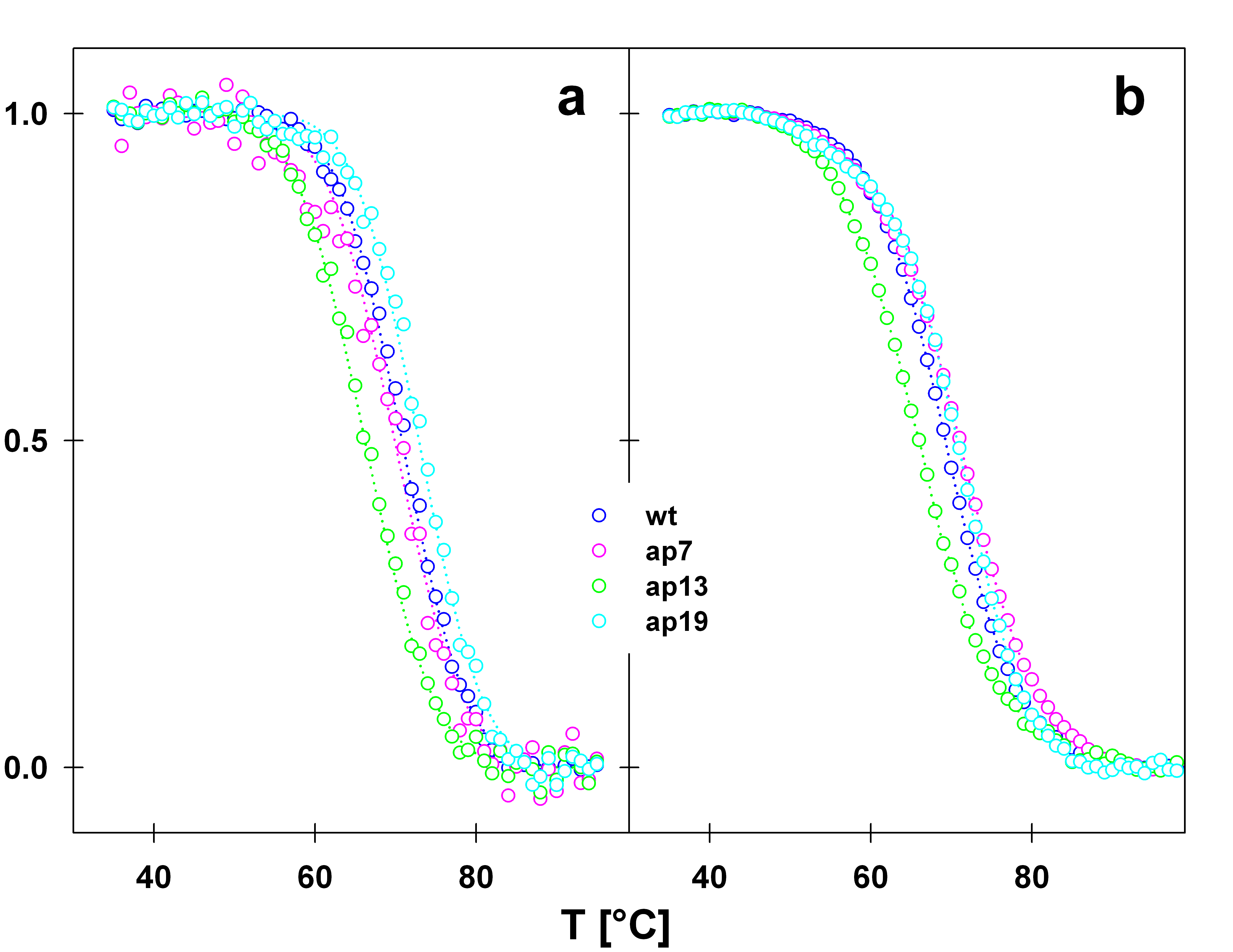


Figure S1: Temperature dependences of the 295 nm UV absorbance value of the *htel-22* quadruplexes measured at (a) 23 μM DNA concentrations in 165 mM K^+^ (10 mM potassium phosphate plus 150 mM KCl), and (b) the NMR samples at ~0.8 mM DNA concentrations in 100 mM K^+^ (20 mM potassium phosphate plus 70 mM KCl).


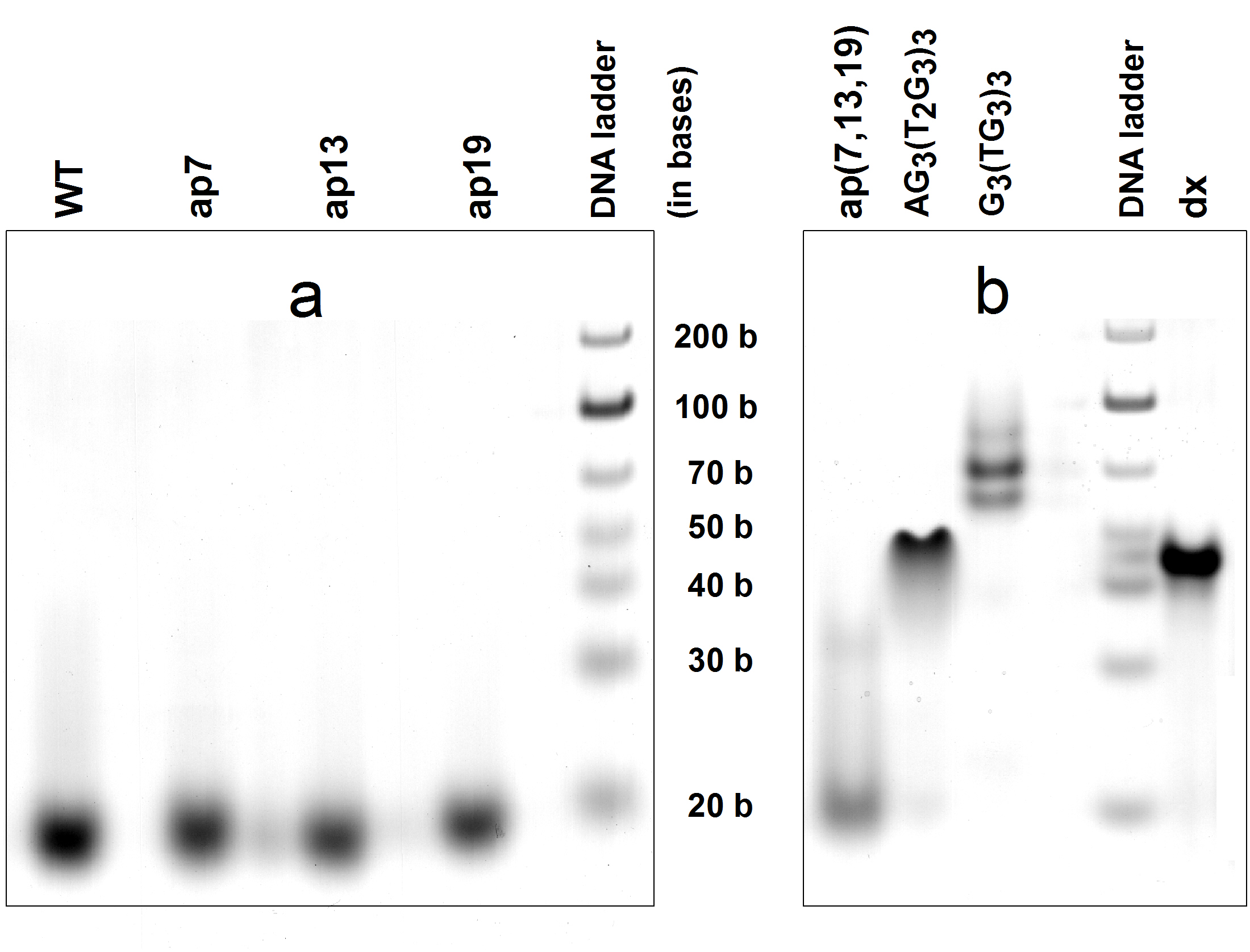


Figure S2: Gel electrophoreses of *htel-22* and of its AP analog quadruplexes performed at 23 μM DNA concentration in (a) 165 mM K^+^, pH 6.8, 23°C, (b) 100 mM K^+^, pH 6.8, 1°C. dx is a duplex of the *wt* sequence with its complementary (C_3_TAA) _3_C_3_T strand.


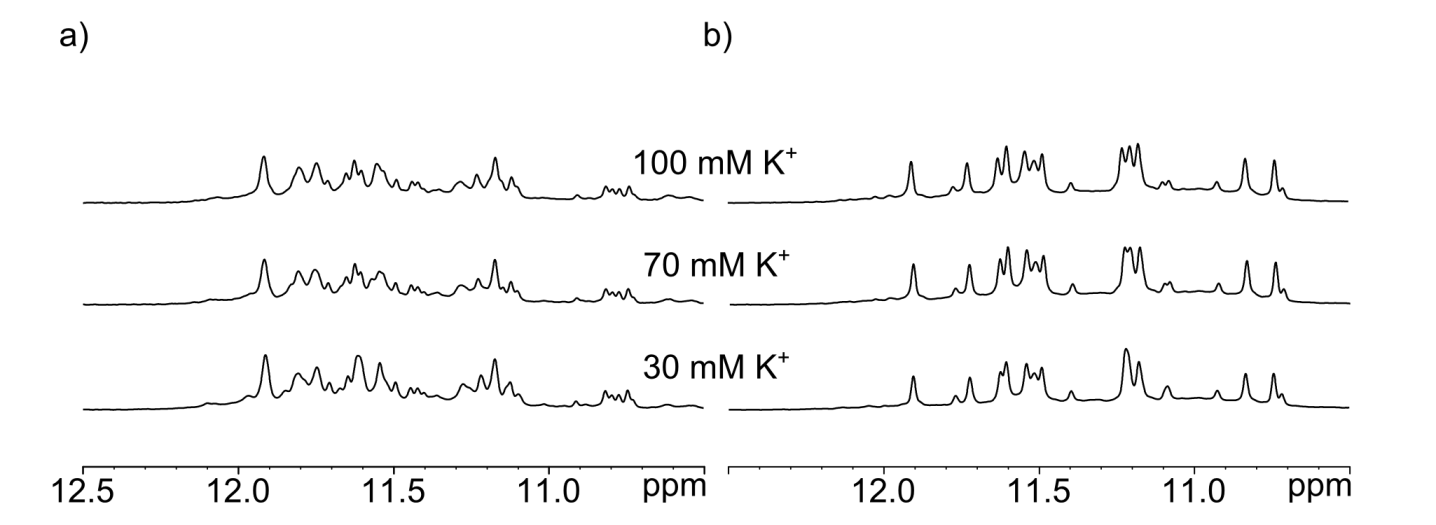


Figure S3: Imino proton regions of ^1^H NMR spectra of (a) *htel-22wt* and (b) *ap19* at three different concentrations of K^+^ ions. The spectra were recorded at 25°C and pH 7.


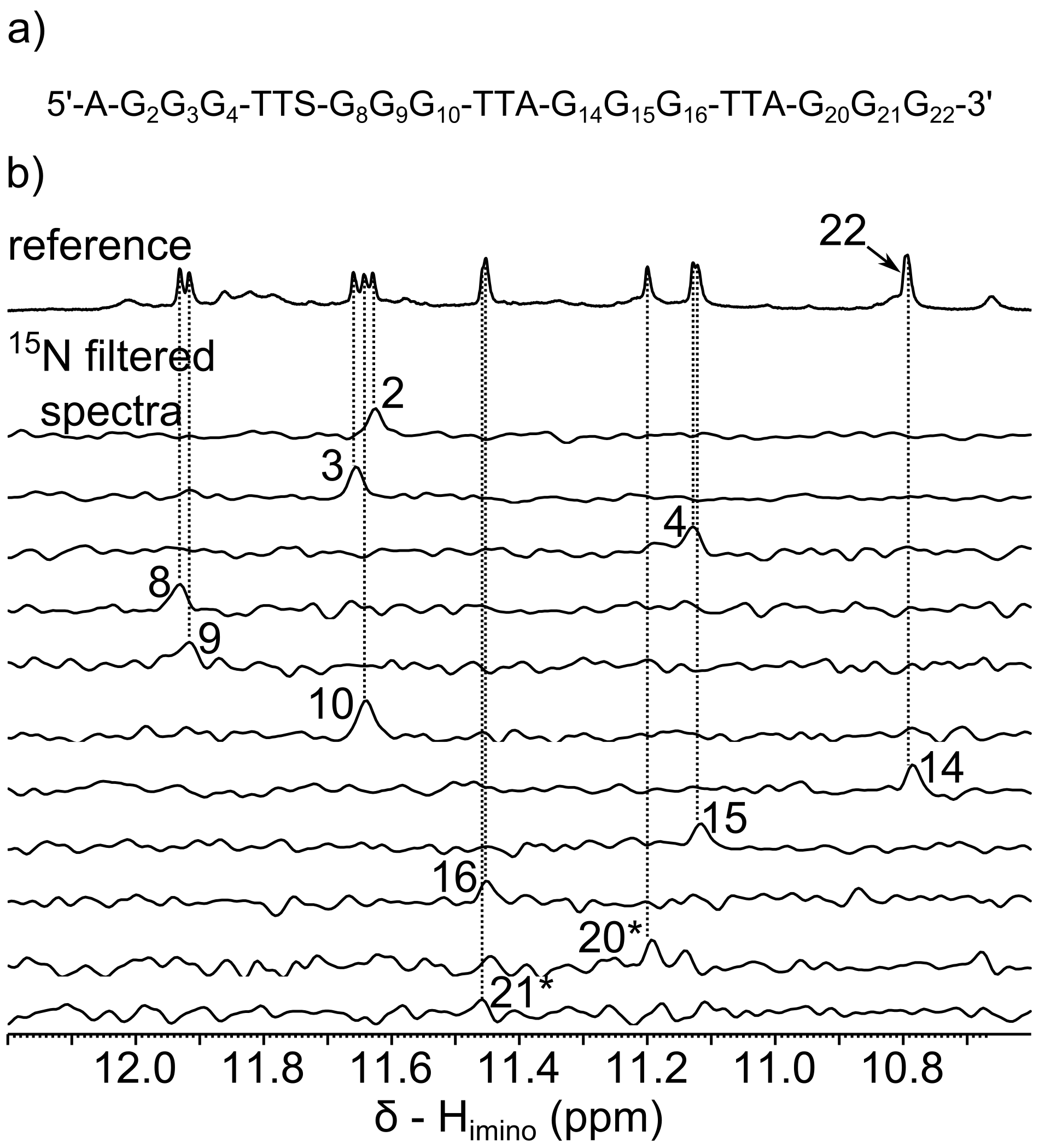


Figure S4. (a) sequence of *ap7* with numbered guanine residues. Letter S denotes the position of the AP site, (b) Reference proton 1D spectrum of *ap7* and ^15^N-filtered spectra of site-specifically enriched samples used for unambiguous assignment of guanine imino proton resonances. Resonances marked with asterisk were not observed directly in the 1D spectra and were assigned in the 2D HNC spectra (see Figure S6). The spectra were recorded at 25°C, pH 6.8, and 30 mM K^+^ concentration.


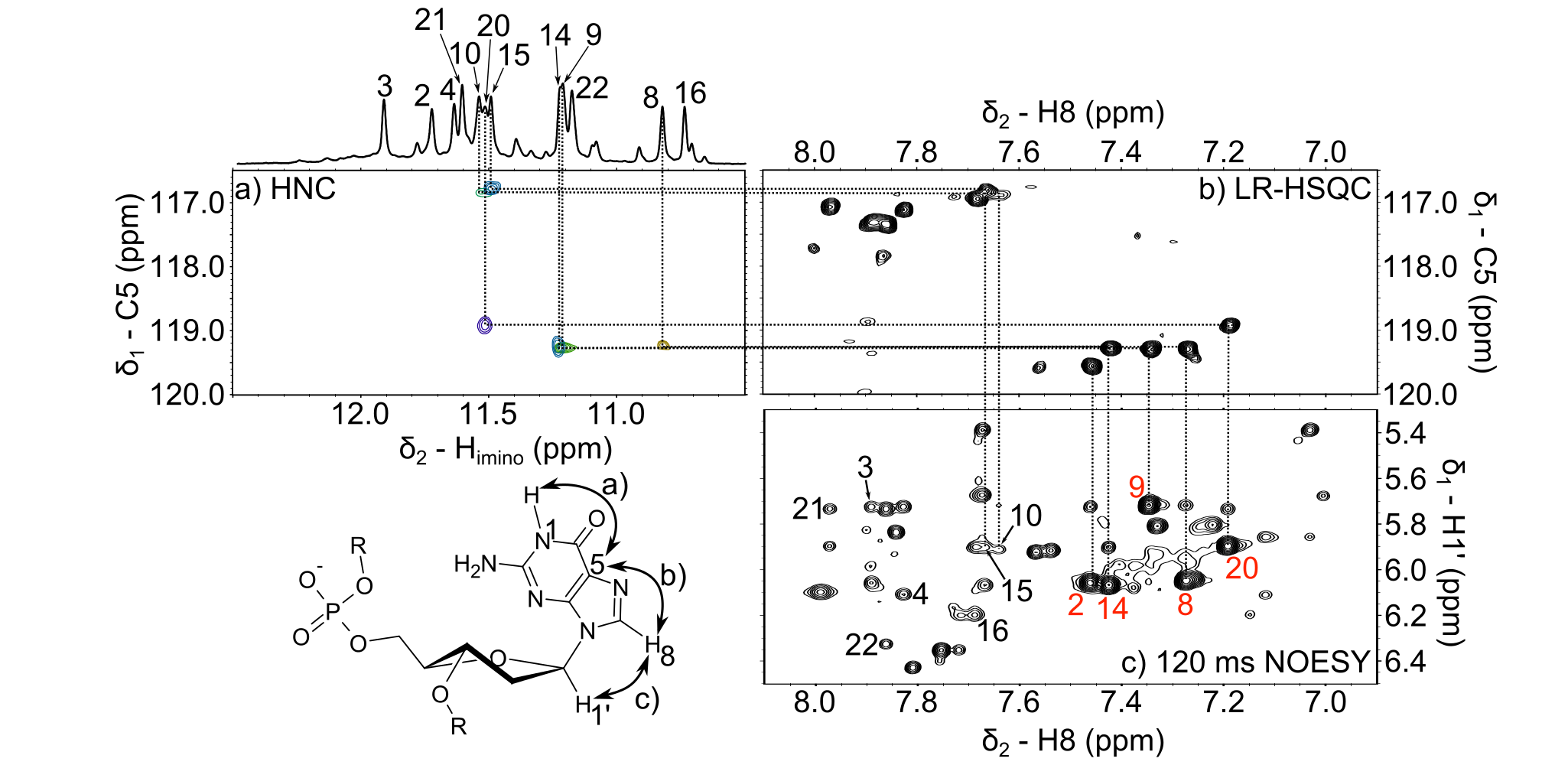


Figure S5. Complete assignment of H8 and H1’ protons in guanine residues of *ap7* (see Supplementary Figure S4a for the oligonucleotide sequence) *via* heteronuclear correlations to imino protons and NOESY spectra. The numbers in c) denote the assignment of intraresidual H1’-H8 crosspeaks to individual guanosines. Guanosine residues in *syn-* conformation are labeled in red. Spectra were recorded at 25°C, pH value of 6.8, and 30 mM K^+^ concentration.


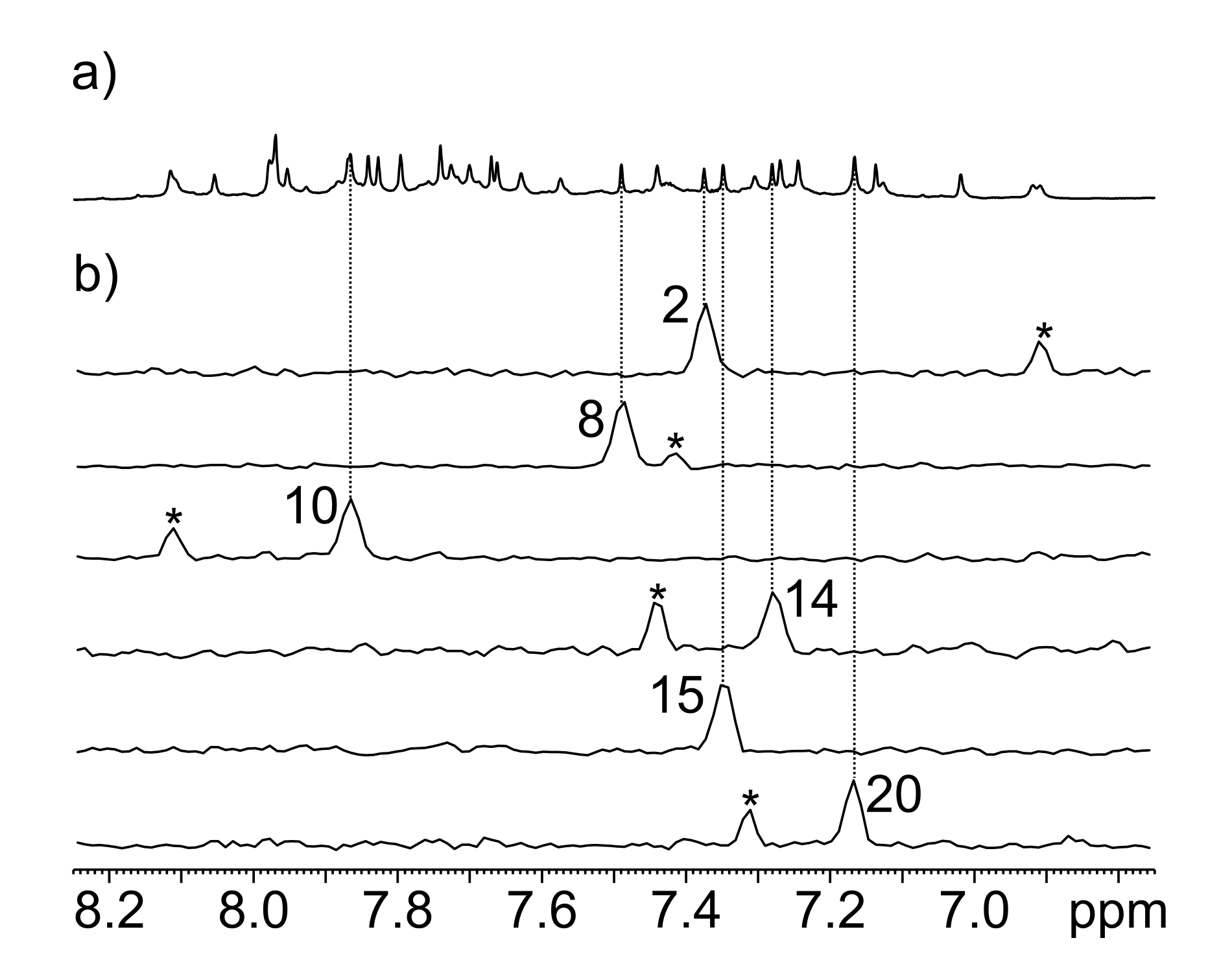


Figure S6. a) aromatic (H6/H8) region of proton 1D spectrum of *ap7* (see Supplementary Figure S4a for the oligonucleotide sequence), b) 1D projections of selective ^1^H-^15^N LR-HSQC spectra measured to confirm assignment of H8 protons in selected guanine residues. The signals marked with asterisks belong to a minor conformation of *ap7* observed in spectra. Spectra were recorded at 25°C, pH value of 6.8, and 30 mM K^+^ concentration.


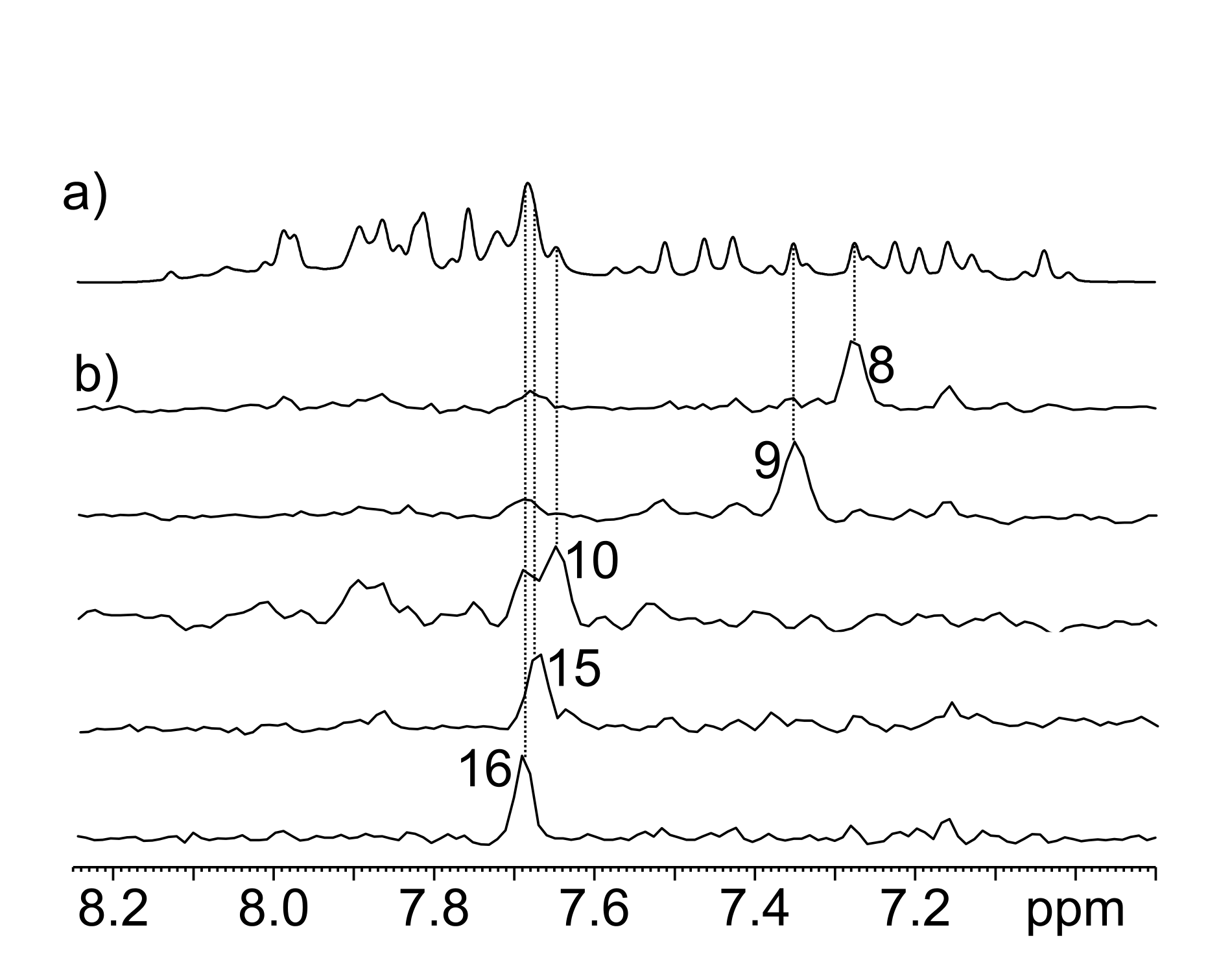


Figure S7. a) aromatic (H6/H8) region of proton 1D spectrum of *ap19* (see Figure 4 for the oligonucleotide sequence), b) 1D projections of selective ^1^H-^15^N LR-HSQC spectra measured to confirm assignment of H8 protons in selected guanine residues. Spectra were recorded at 25°C, pH value of 6.8, and 30 mM K^+^ concentration.


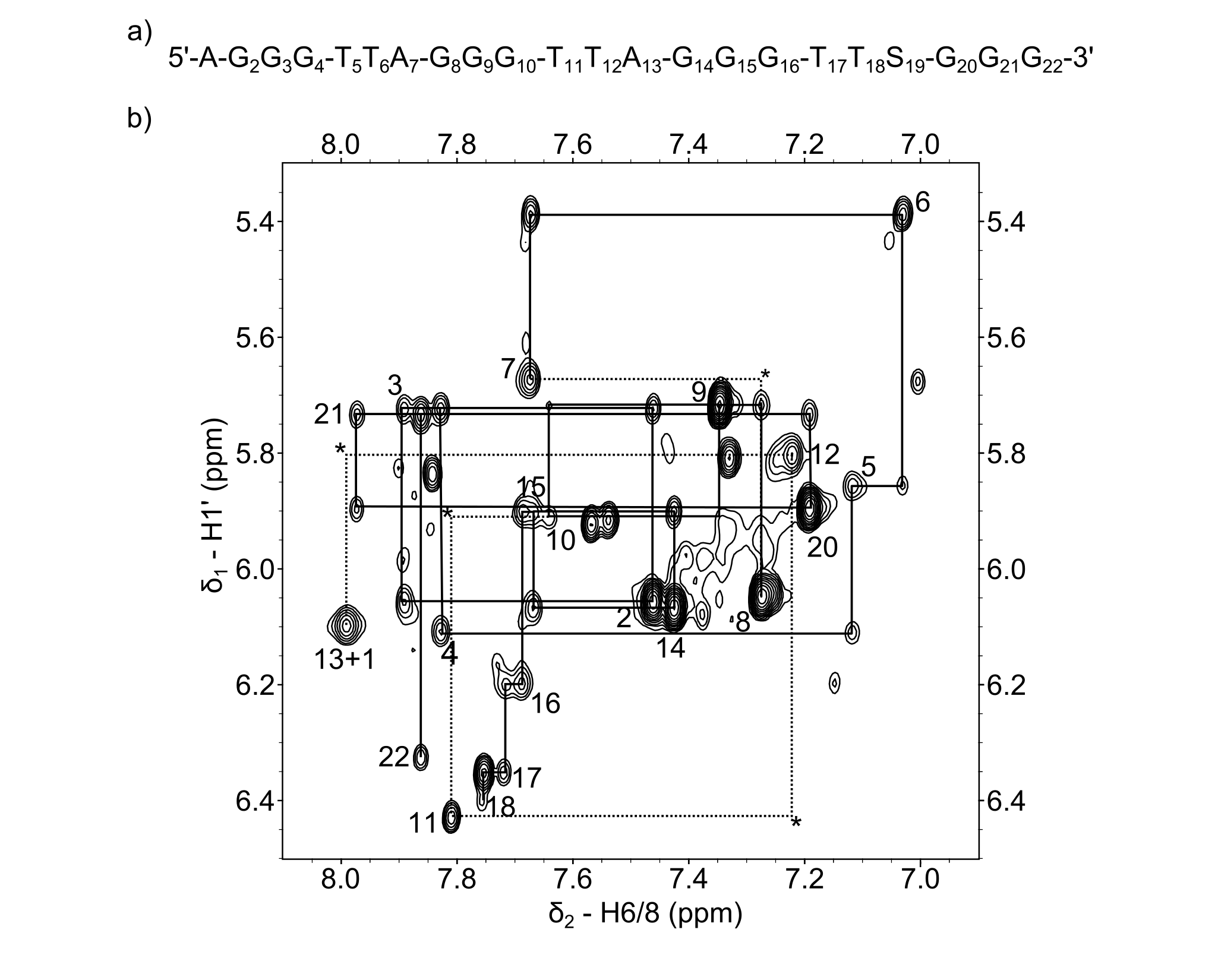


Figure S8. (a) oligonucleotide sequence of *ap19* (S = dSpacer), (b) sequential connectivity between aromatic and H1’ protons in 150 ms NOESY spectrum of *ap19*. Solid lines represent a path where sequential H1’-H8 crosspeaks are well resolved and visible. Dashed lines and asterisks denote a broken connectivity with sequential crosspeaks either weak or missing. The spectrum was recorded at 25°C, pH 6.8 and 100 mM K^+^ concentration.


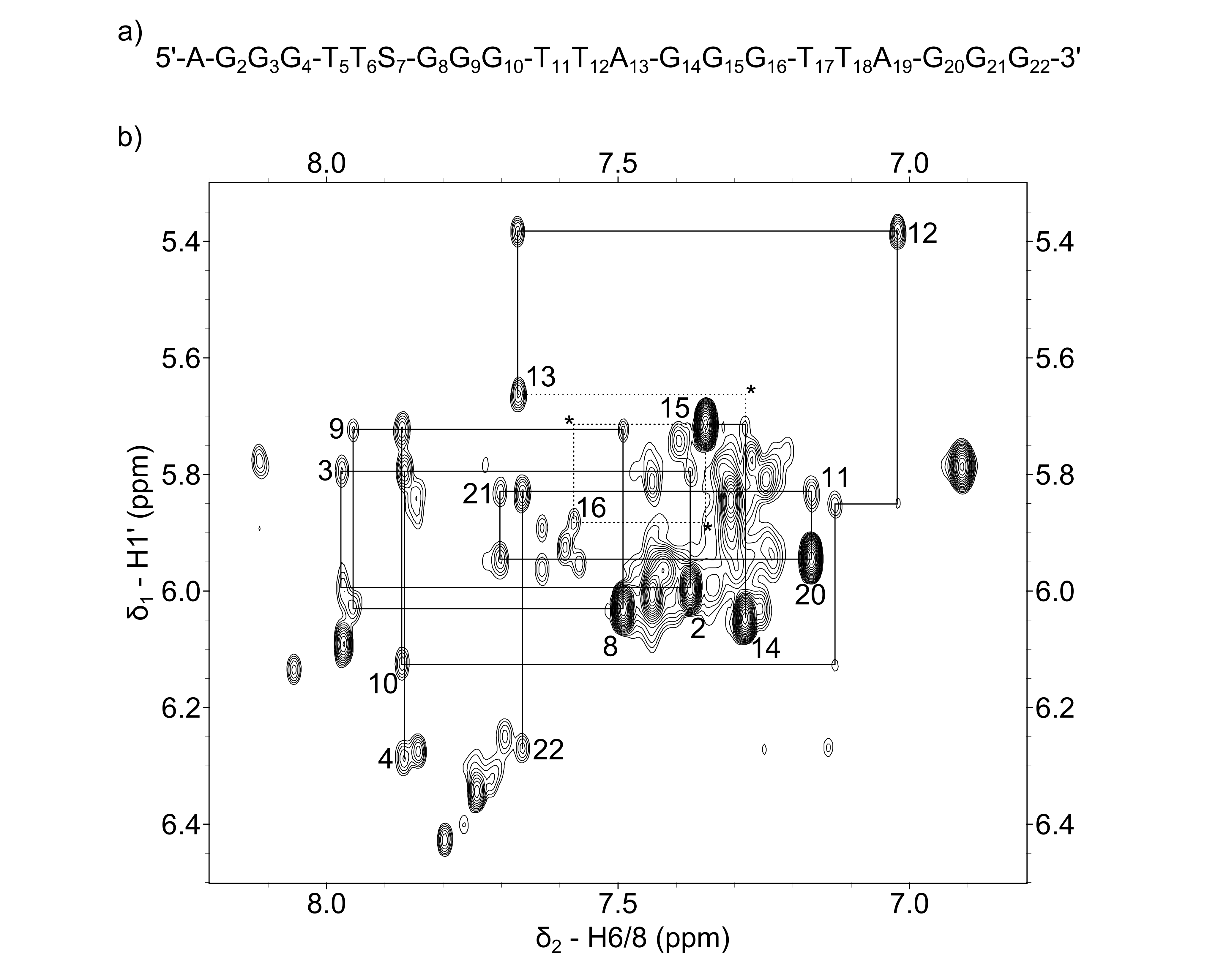


Figure S9. (a) oligonucleotide sequence of *ap7* (S = dSpacer), (b) portion of 150 ms NOESY spectrum of *ap7* showing the sequential connectivity between H1’ and aromatic protons of guanines and bases located in the middle edgewise loop. Other residues were not assigned due to missing connectivity and spectral overlap. Dashed lines and asterisks denote connectivity interrupted at *anti*-/*syn*- steps. The spectrum was recorded at 25°C, pH 6.8 and 100 mM K^+^ concentration.

*
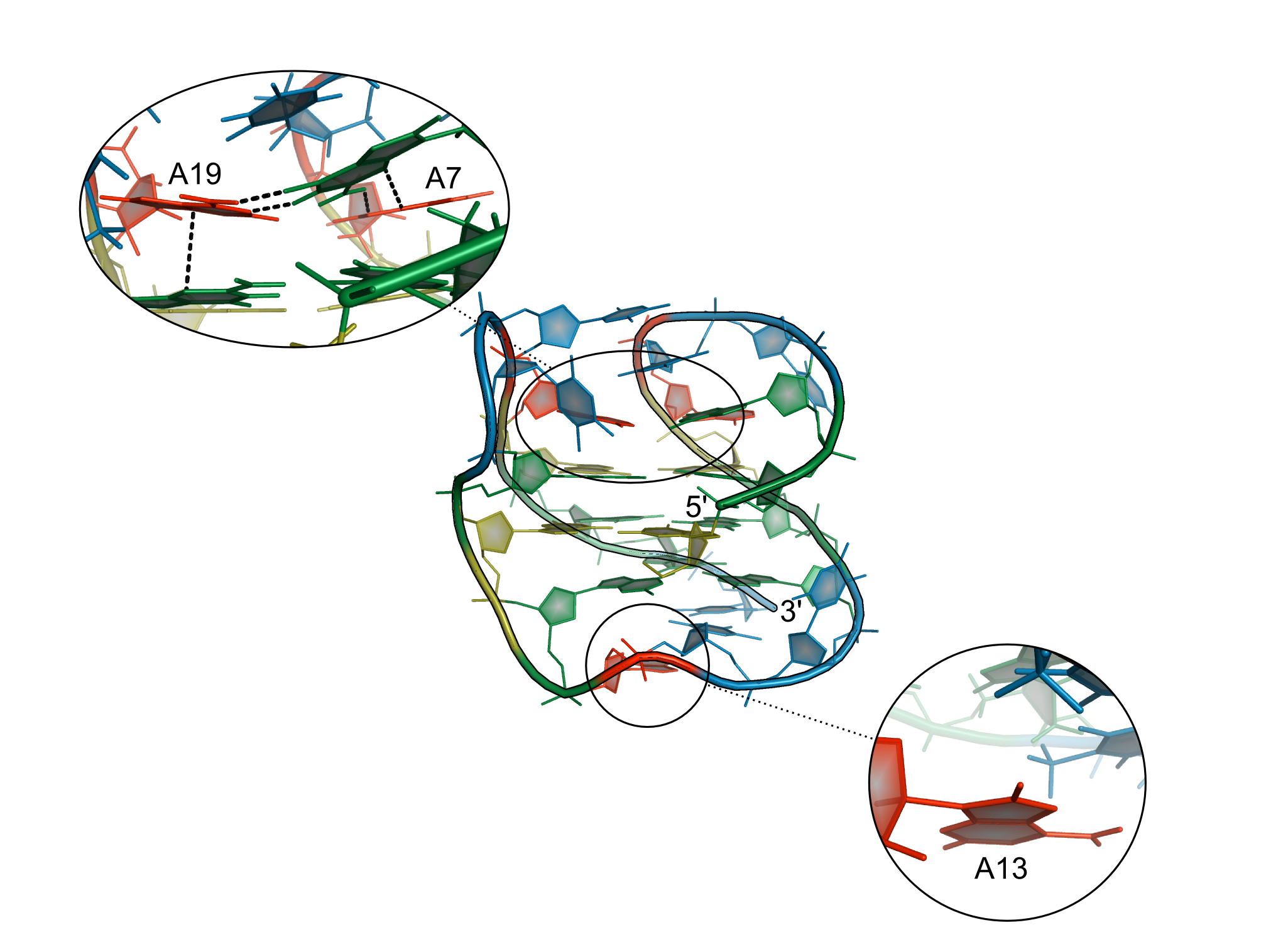
*

Figure S10. Structure of K^+^-stabilized 2-tetrad antiparallel basket (PDB ID *2kf8*) quadruplex topology adopted by a fragment of the human telomeric sequence with highlighted positions and interactions of loop adenines. The adenosine numbering corresponds to the position of these residues in *htel-22wt* sequence. Adenosine residues are colored red, thymidine residues are colored blue, and guanosine residues in *syn-* and *anti-*conformation are colored green and dark yellow, respectively. Black dashed lines represent the directions of stabilizing hydrogen bonding and stacking interactions.
